# MiR‐563 restrains cell proliferation via targeting LIN28B in human lung cancer

**DOI:** 10.1111/1759-7714.13257

**Published:** 2019-11-25

**Authors:** Xuefei Zhang, Mo Li, Ge Sun, Yu Bai, Desheng Lv, Changhong Liu

**Affiliations:** ^1^ Department of Thoracic Surgery The Second Hospital of Dalian Medical University Dalian China

**Keywords:** LIN28B, lung cancer, MiR‐563, proliferation

## Abstract

**Background:**

Previous investigations have revealed that miR‐563 is associated with a number of diseases including the ossification of posterior longitudinal ligament, Parkinson's disease or drug resistance to leukemia. Yet, the role of miR‐563 and its molecular mechanism in the initiation and progression of cancers has not been previously explored. In this study, we aimed to provide clues to the function of miR‐563 and its direct target in lung cancer.

**Methods:**

Online informatics software was applied to predict the target genes of miR‐563. MiR‐563 targeting LIN28B was evaluated through the luciferase reporter gene analysis. The effect of miR‐563 on LIN28B at the level of RNA and protein was detected using RT‐PCR and immunoblotting. The ability of proliferation of human lung cancer A549 was examined by MTT assay. RNA interference targeting LIN28B was examined through immunoblotting. The level of miR‐563 and LIN28B and their correlation were analyzed in 27 cases of lung tumor tissues by real‐time PCR.

**Results:**

Oncogenic LIN28B was identified as one of the target genes of miR‐563 in lung cancer cells. MiR‐563 dose‐dependently decreased the LIN28B RNA level and subsequently its protein level in the cells. Cell proliferation was suppressed by ectopic miR‐563 expression and was accelerated after endogenous miR‐563 was knocked down by its inhibitor. However, silence in LIN28B reversed promotion of cell proliferation by the inhibition of miR‐563. In lung cancer tissues, miR‐563 was decreased and negative correlation of miR‐563 and LIN28B was shown.

**Conclusion:**

MiR‐563 plays a tumor suppressive role in lung cancer progression via targeting oncogenic LIN28B.

## Introduction

Lung cancer among people worldwide still remains the leading cause of cancer‐related death and more than 80% patients with lung cancer are diagnosed with non‐small cell lung cancer (NSCLC).^1^, ^2^ As one of the conserved non‐coding RNAs, microRNAs (miRNAs) with around 22 nucleotides can function in modulating the expression of their target genes through directly binding to the 3′untranslated regions (3′UTR) of mRNAs of their target genes and then degrading mRNA or suppressing translation.^3^, ^4^, ^5^ MiRNAs have been revealed to be associated with a great sea of processes in cells such as apoptosis, proliferation, migration or differentiation.^6^, ^7^, ^8^ MiRNAs can function as either oncogenes or tumor suppressors and play important roles in the initiation and progression in tumorigenesis.^9^ Evidence in the report by Cao *et al*. revealed a decrease in miR‐563 level in adriamycin (ADM)‐resistant leukemia cells.^10^ Some miRNAs including miR‐563 with a high degree are related to the pathogenesis of Parkinson's disease.^11^ MiR‐563 plays a promoting role via directly targeting SMURF1 and can function as a circulating biomarker in the osteogenic differentiation of the posterior longitudinal ligament.^12^, ^13^ However, the role of miR‐563 and its molecular mechanism in the development of any type of cancers has not been previously reported.

Both LIN28B and LIN28A from the LIN28 protein family function as the RNA‐binding protein in the cells.^14^ Highly expressed LIN28 is firstly revealed in liver cancer tissues.^15^ In addition to liver cancer, LIN28B has also been found to be elevated in neuroblastoma, lung cancer, pancreatic cancer, and colorectal cancer.^16^, ^17^, ^18^, ^19^ Yet, the association of LIN28B with miR‐563 in any type of cancer remains unexplored.

In our study, we identified miR‐563 as a new tumor suppressor in lung cancer. MiR‐563 can target the LIN28B mRNA 3′UTR to restrain the expression of LIN28B in lung cancer cells. The miR‐563/LIN28B signaling regulates the cell growth in lung cancer. A negative correlation exists between miR‐563 and LIN28B in clinical lung cancer samples. Our finding provides new insight into the role of miR‐563 and its target in the progression of lung cancer.

## Methods

### Cell culture

Human lung cancer cell line A549 was obtained from the American Type Culture Collection (ATCC, USA), cultured in RPMI‐1640 medium (Invitrogen, USA) with 10% fetal bovine serum and maintained in a humidified incubator at 37°C with 5% CO_2_.

### MiRNAs and siRNAs

MiRNAs and siRNAs including miR‐563, anti‐miR‐563 and two siRNAs (si‐LIN28B‐1 and si‐LIN28B‐2) were synthesized by RiboBio (Guangzhou, China). Lipofectamine 2000 (Invitrogen, USA) was used to transfect miRNAs and siRNAs including miR‐563, anti‐miR‐563 or si‐LIN28B‐1/2 into lung cancer A549 cells at a concentration of 50 nM, 100 nM or 200 nM.

### Cell proliferation

The proliferation ability of lung cancer cells was analyzed by 3‐(4, 5‐dimethylthiazol‐2‐yl)‐2, 5‐diphenyltetrazolium bromide (MTT) assay according to the manufacturer's instructions. After the transfection of miR‐563, anti‐miR‐563 or si‐LIN28B‐2 into A549, the cells were transferred to 96‐well plates. At the indicated time point (0 hours, 24 hours, 48 hours or 72 hours), MTT solution (5 mg/mL) was placed into every well of 96‐well plates and incubated for more four hours. The medium was discarded and 100 μL DMSO (per well) was added to dissolve the formosan. A microplate reader was used to examine the absorbance values at 490 nm.

### Immunoblotting

At the 48 hours of transfection, lung cancer cells were lyzed using RIPA buffer, and the cellular protein samples were separated on 15% SDS‐PAGE. The protein samples were shifted from SDS‐PAGE gel to PVDF membranes (Thermo Fisher Scientific, USA). The primary antibodies including anti‐LIN28B (Abcam, USA) or anti‐β‐actin (Abcam, USA) were used to incubate with the membranes.

### RNA preparation, RT‐PCR and real‐time PCR

TRIzol reagent from Invitrogen Incorporation (USA) was applied to extract total RNA in cells and lung cancer tissues following the manufacturer's instructions. First strand cDNA synthesis was performed using EasyScript First‐Strand cDNA Synthesis SuperMix (TransGen Biotech, China) to test the expression of LIN28B in cells and tissues. For real‐time PCR assay, TransStart Top Green qPCR SuperMix (TransGen Biotech, China) was utilized. For the detection of miR‐563, total RNA was polyadenylated through poly (A) polymerase (Ambion, USA). The level of miR‐563 or LIN28B in cells and tissues were normalized by the internal loading control, U6 or GAPDH. All PCR reactions were repeated at least three times.

### Luciferase reporter gene analysis

The wild‐type LIN28B 3′UTR with the binding site of miR‐563 or the mutant LIN28B 3′UTR with five substitutions of the predicted binding site of miR‐563 was cloned into pGL3‐Control vector (Promega, USA). The cells were seeded into 24‐well plates and then transfected with miR‐563, anti‐miR‐563, or negative control (NC) and constructed reporter vectors. The Dual‐Luciferase Reporter Assay System (Promega, USA) was utilized to test the luciferase activities of the cells 48 hours post‐transfection.

### Statistical analysis

GraphPad Prism software (GraphPad Software, Inc., USA) was utilized to perform the statistical analysis. All data are shown as the mean values with standard error of the mean (SEM). A *P*‐value less than 0.01 was statistically significant. A Student's *t*‐test was used for the statistical analysis of two groups. For the significant comparison of more than two groups, one‐way analysis of variance (ANOVA) was applied. The correlation of miR‐563 and LIN28B in clinical lung cancer samples was analyzed by Pearson's correlation coefficient.

## Results

### MiR‐563 can bind to and directly target oncogenic LIN28B in lung cancer cells

**Figure 1:**
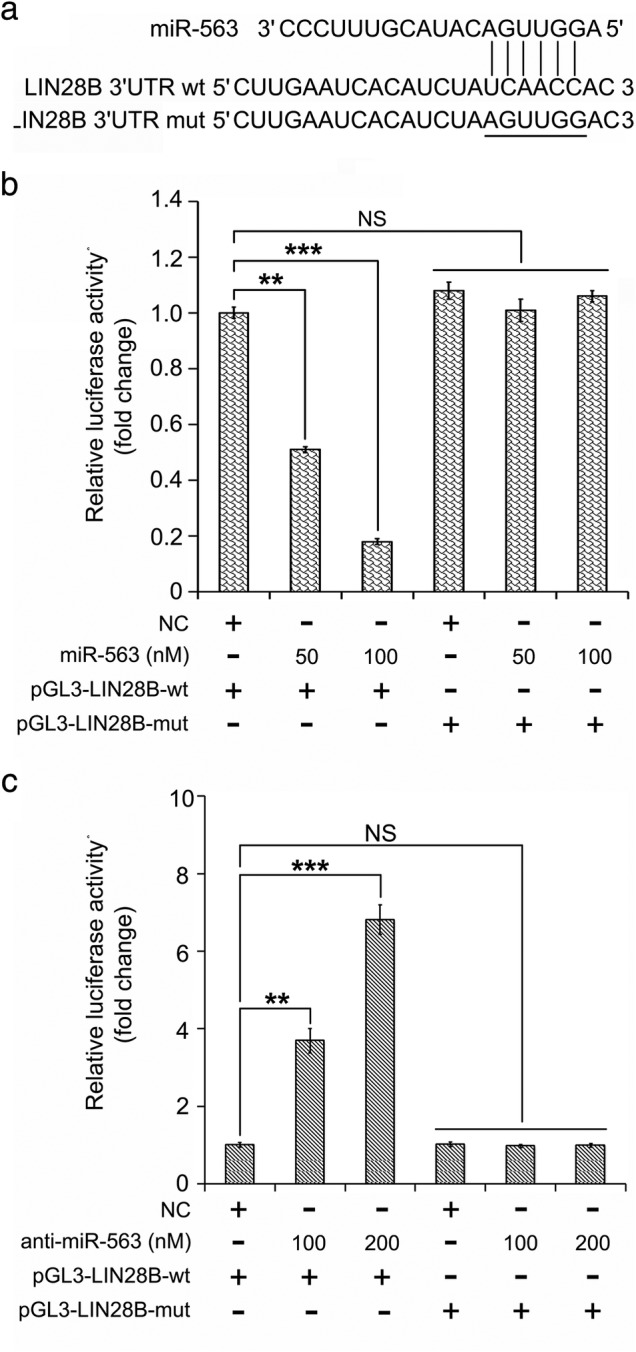
MiR‐563 can bind to and directly target oncogenic LIN28B in lung cancer cells. (**a**) Wild‐type (wt) and mutant type (mut) 3′UTR of LIN28B mRNA containing the binding site of miR‐563 through Targetscan prediction (http://www.targetscan.org). (**b,c**) The binding of miR‐563 or anti‐miR‐563 to 3′UTR of LIN28B mRNA was tested through the luciferase reporter gene assay in lung cancer cells. NS, not significant; **P < 0.01, ***P < 0.001.

It has not been previously reported that miR‐563 is associated with some diseases including posterior longitudinal ligament, Parkinson's disease or drug resistance to leukemia. However, its role during the development of any kind of tumor has not been reported. Therefore, we were curious to analyze the function of miR‐563 and its target gene in lung cancer. First, we predicted the targets of miR‐563 through TargetScan (http://www.targetscan.org/). We were very interested to learn of the important role of LIN28B in carcinogenesis. We cloned the wild‐type 3′UTR of LIN28B mRNA (wt) containing a putative binding site with the seed region of miR‐563 and the mutant 3′UTR of LIN28B mRNA with five substitutions of the predicted binding site of miR‐563 (mut) into luciferase vector (Fig 1a). A luciferase reporter gene analysis was performed to test whether miR‐563 could bind to the 3′UTR of LIN28B mRNA. We found that ectopic miR‐563 expression at the elevated concentration (50 and 100 nM) in lung cancer A549 cells was able to obviously induce the decrease in the luciferase activities of wt (wild‐type) vectors of 3′UTR of LIN28B. Nevertheless, miR‐563 lost the control of luciferase vectors of 3′UTR of LIN28B when the mutant (mut) form was introduced into lung cancer cells (Fig 1b). When the inhibitor of miR‐563 (anti‐miR‐563) was introduced into the cells, the luciferase activities of wt vectors of 3′UTR of LIN28B were induced and mut vectors of 3′UTR of LIN28B cannot be affected (Fig 1c). All our findings implied that miR‐563 was capable of directly targeting oncogenic LIN28B in lung cancer.

### MiR‐563 controls the LIN28B expression in lung cancer cells

**Figure 2:**
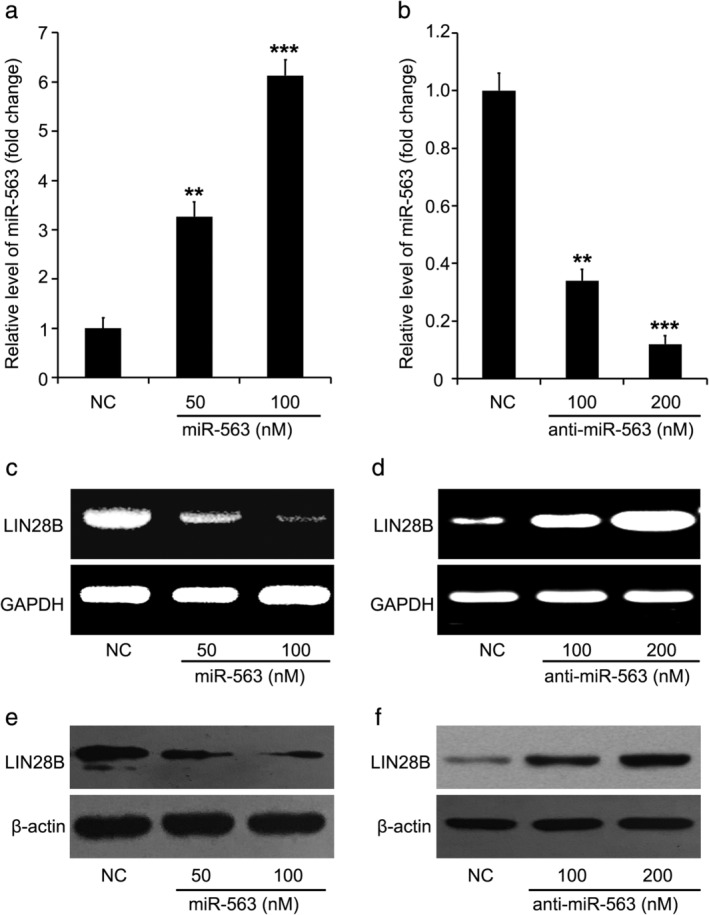
MiR‐563 controls the LIN28B expression in lung cancer cells. (**a,b**) The level of miR‐563 in miR‐563 or its inhibitor (anti‐miR‐563)‐transfected A549 cells was evaluated. (**c,d**) The RNA level of LIN28B in miR‐563 or its inhibitor (anti‐miR‐563)‐transfected A549 cells was detected. (**e,f**) The protein level of LIN28B in miR‐563 or its inhibitor (anti‐miR‐563)‐transfected A549 cells was detected.

During further investigation, we aimed to reveal the details by which miR‐563 regulates the expression of LIN28B through detecting the level of mRNA and protein by RT‐PCR and immunoblotting when miR‐563 is overexpressed or inhibited. Lung cancer cell line A549 was treated with a different dose of miR‐563 or its inhibitor (50, 100 or 200 nM) for overexpression or knockdown of miR‐563. The transfection efficiency of miR‐563 and its inhibitor (anti‐miR‐563) was analyzed through quantitative real‐time PCR (Fig 2a,b). We found that miR‐563 introduction markedly decreased the RNA level of LIN28B (Fig 2c). MiR‐563 silenced by its inhibitor, anti‐miR‐563, significantly induced the LIN28B expression in the cells (Fig 2d). MiR‐563 was then able to downregulate the protein level of LIN28B in the cells (Fig 2e). Meanwhile, miR‐563 silenced by anti‐miR‐563 induced the protein level of LIN28B in the cells (Fig 2f). All these findings indicated that the expression of LIN28B was restrained by miR‐563 in lung cancer cells.

### MiR‐563 depresses cell proliferation of lung cancer via targeting LIN28B

**Figure 3:**
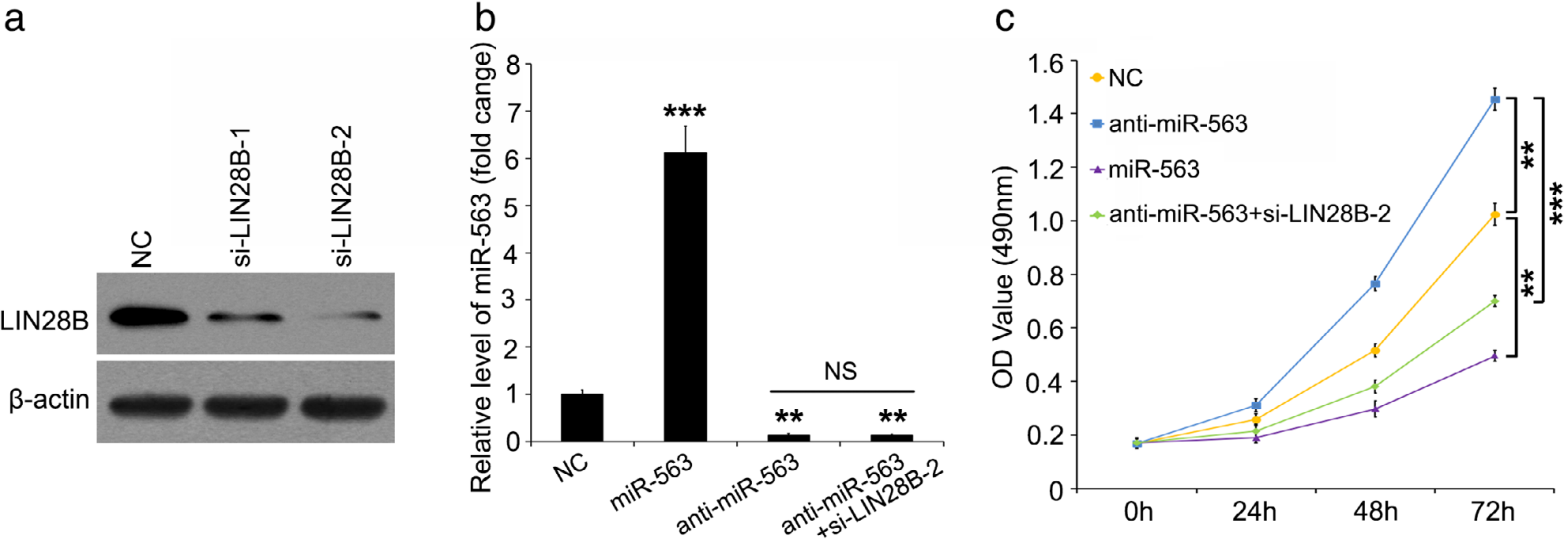
MiR‐563 depresses cell proliferation of lung cancer via LIN28B. (**a**) The knockdown efficiency of si‐LIN28B‐1 and si‐LIN28B‐2 were examined by immunoblotting. (**b**) The transfection efficiency of miR‐563 (or anti‐ miR‐563) was confirmed by quantitative real‐time PCR. (**c**) The effect of miR‐563, anti‐miR‐563 and/or si‐LIN28B‐2 on cell proliferation was analyzed by MTT assay. **P < 0.01, ***P < 0.001.

To better illustrate the role of miR‐563 in lung cancer progression, we transfected miR‐563, anti‐miR‐563 and/or small interference RNAs targeting LIN28B mRNA into lung cancer cells and then analyzed the cell proliferation. Immunoblotting analysis confirmed that two different siRNAs targeting LIN28B mRNA (si‐LIN28B‐1 and si‐LIN28B‐2) could both effectively induce knockdown of LIN28B in lung cancer cells, and the interference effect of si‐LIN28B‐2 was more obvious than that of si‐LIN28B‐1 (Fig 3a). Quantitative real‐time PCR assay showed the level of miR‐563 after the cells were treated with miR‐563 or anti‐miR‐563 (Fig 3b). As expected, compared with the negative control (NC) group, miR‐563 overexpression significantly repressed the cell proliferation of lung cancer and then silencing miR‐563 mediated by anti‐miR‐563 accelerated the lung cancer cell proliferation (Fig 3c). To further confirm the function of LIN28B in miR‐563‐regulated cell proliferation in lung cancer, we disturbed the LIN28B expression in anti‐miR‐563‐contained lung cancer cells. We found that by silencing LIN28B we were able to destroy the anti‐miR‐563‐accelerated cell proliferation in lung cancer (Fig 3c). Collectively, we conclude that miR‐563 restrains lung cancer cell proliferation via inhibition of LIN28B.

### Low miR‐563 is associated with high LIN28B in human lung cancer tissue

**Figure 4:**
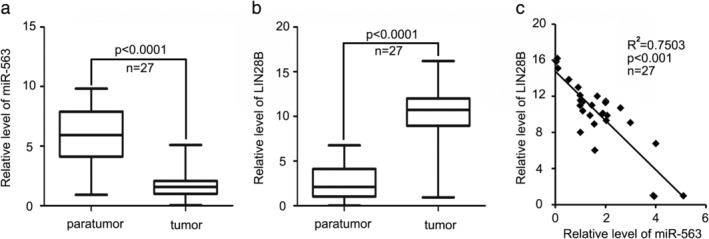
In human lung cancer tissues, low miR‐563 is associated with high LIN28B. (**a,b**) The level of miR‐563 or LIN28B in 27 cases of lung tumor tissues and their paratumor tissues was evaluated through real‐time PCR analysis. (**c**) Real‐time PCR assay and Pearson's *r* analysis was used to analyze the level of miR‐563 and LIN28B and their relationship. R^2^ = 0.7503. ***P < 0.001.

Finally, we tested the level of miR‐563 and LIN28B and analyzed the pathophysiological correlation in 27 paired clinical lung tumor tissues and paratumor tissues by quantitative real‐time PCR assay. Consistent with the tumor suppressive role of miR‐563 in the above findings, the level of miR‐563 in tumor tissues was much lower than that in paired paratumor tissues (Fig 4a). We then found that LIN28B was upregulated in 27 cases of human lung cancer tissues (Fig 4b). Our data finally revealed that in 27 cases of lung cancer tissues the level of miR‐563 was negatively related to the level of LIN28B (R^2^ = 0.7503, *P* < 0.001) (Fig 4c). Therefore, we concluded that decreased miR‐563 induced LIN28B expression.

## Discussion

MiRNAs are reported to play a great part in cancer initiation or progression.^20^, ^21^, ^22^ Evidence in the report by Cao *et al*. revealed a decrease in miR‐563 level in adriamycin‐resistant leukemia cells.^10^ Some miRNAs including miR‐563 to a high degree are related to the pathogenesis of Parkinson's disease.^11^ MiR‐563 plays a promoting role via directly targeting SMURF1 and can function as a circulating biomarker in the osteogenic differentiation of posterior longitudinal ligament.^12^, ^13^ However, the role of miR‐563 and its molecular mechanism in the development of any type of cancer has to date not been reported.

In the present investigation, using online informatics software we found that oncogenic LIN28B was one of many predicted target genes of miR‐563. Yet, the association between miR‐563 and LIN28B remains unreported. Accordingly, we were interested in whether miR‐563 could target oncogenic LIN28B to affect the development of lung cancer. Based on the cloning of luciferase vector containing wild‐type or mutant type 3′UTR of LIN28B mRNA with the binding sites of miR‐563, we tested the binding of miR‐563 to 3′UTR of LIN28B mRNA through luciferase reporter gene analysis. Notably, we found that that miR‐563 was able to directly bind to 3′UTR of LIN28B mRNA and decrease its luciferase activities but not the mutated vectors. Furthermore, our results confirmed that miR‐563 was able to repress the level of RNA and protein of LIN28B in lung cancer cells. For the investigation of the function of miR‐563 in lung cancer, we observed that miR‐563 played a tumor suppressive role in the cell proliferation of lung cancer. However, there was an obvious induction of cell proliferation in miR‐563‐silenced lung cancer cells. Taking this a step further, the role of miR‐563/LIN28B signaling was evaluated. Interestingly, the cell proliferation driven by anti‐miR‐563 could be disturbed by the silencing of LIN28B. In the study of clinical lung cancer patient samples, the axis of miR‐563/LIN28B was confirmed and it is consistent with all the above findings in lung cancer cells.

Both LIN28B and LIN28A from the LIN28 protein family function as RNA binding proteins in cells.^14^ Highly expressed LIN28 was first revealed in liver cancer tissues.^15^ Besides liver cancer, in neuroblastoma, lung cancer, pancreatic cancer, or colorectal cancer, LIN28B has also been found to be elevated.^16^, ^17^, ^18^, ^19^ In the present investigation, we revealed that LIN28B served as one target gene of miR‐563 in lung cancer. In clinical lung cancer tissues, miR‐563 was downregulated and its target, LIN28B was upregulated. When miR‐563 was decreased by its inhibitor, cell proliferation was accelerated. However, the expression of LIN28 was silenced in the promotion of cell proliferation. All these findings from lung cancer cells and clinical lung cancer tissues verify each other.

In summary, here we provide a novel role of miR‐563 in lung cancer growth via targeting LIN28B. MiR‐563 can directly bind to the 3′UTR of LIN28B mRNA and repress its expression in lung cancer cells. The axis of miR‐563/LIN28B plays a key role in lung cancer cell growth. In clinical lung cancer samples, miR‐563 is negatively associated with LIN28B. Therapeutically, miR‐563 has been shown to be a potential target in the treatment of lung cancer and could be a promising prognostic marker for lung cancer in the future.

## Disclosure

The authors declare that they have no conflicts of interest.
